# Alcohol and the Cerebellum: Effects on Balance, Motor Coordination, and Cognition

**Published:** 1995

**Authors:** Edith V. Sullivan, Margaret J. Rosenbloom, Anjali Deshmukh, John E. Desmond, Adolf Pfefferbaum

**Affiliations:** Edith V. Sullivan, Ph.D., is an associate professor in the Department of Psychiatry and Behavioral Sciences, Stanford University School of Medicine, Stanford, CA, and a health science specialist in the Psychiatry Service, Veterans Affairs (VA) Palo Alto Health Care System, Palo Alto, California. Margaret J. Rosenbloom, M.A., is a health science specialist in the Psychiatry Service, VA Palo Alto Health Care System. Anjali Deshmukh, M.D., is a postdoctoral fellow in the Psychiatry Service, VA Palo Alto Health Care System. John E. Desmond, Ph.D., is a research fellow in the Department of Radiology, Stanford University. Adolf Pfefferbaum, M.D., is a professor in the Department of Psychiatry and Behavioral Sciences, Stanford University School of Medicine, and director of psychiatry research in the Psychiatry Service, VA Palo Alto Health Care System

**Keywords:** AODE (alcohol and other drug effects), cerebellum, motor coordination, cognitive process, equilibrium, brain atrophy, AOD impairment

## Abstract

Alcoholics often suffer from motor incoordination resulting from alcohol-related cerebellar damage. However, the effect of cerebellar structural damage on cognitive functioning has not been clearly demonstrated. It is not known if the relationships observed between cerebellar damage and functional impairments persist with abstinence from alcohol. Cell death may cause permanent loss of function, whereas tissue shrinkage without permanent cell loss might represent the potential for recovery. This article examines research on the interrelationship of alcohol-related abnormalities in cerebellar structure and function. Research such as this may provide knowledge to guide future rehabilitation efforts.

One of the most widely recognized signs of acute alcohol intoxication is staggering gait. The motor incoordination element of intoxication is routinely assessed by highway patrol officers when they use standard tests of stance imbalance (otherwise known as gait ataxia) and eye-hand incoordination.

The obvious signs of gait and coordination disturbance demonstrated by these simple tests usually resolve once the person has become sober. However, careful study of alcoholics^1^ reveals residual, subtle, persistent deficits in balance that may put them at increased risk of accidents, such as falling. These deficits may be related to alcohol-induced pathological changes in the cerebellum, one of the brain’s main centers of postural control and motor coordination.

In addition to its role in controlling movement, the cerebellum may play an important role in the acquisition of motor skills and the cognitive processes that control movement (Ito 1993). The cerebellum also may help augment cognitive processes originating in the cerebrum (Leiner et al. 1993), such as language production (Appollonio et al. 1993; Petersen 1993) and mental imagery (Ryding et al. 1993). The role of the cerebellum in cognition is complex and controversial.

This article reviews current knowledge on the motor and cognitive processes controlled or facilitated by the cerebellum and describes damage to the cerebellum associated with long-term alcoholism. The article then explains how researchers are seeking to relate this damage both to problems with balance and motor coordination and to some of the characteristic cognitive deficits associated with alcoholism.

## Structure of the Cerebellum

**Figure 1:**
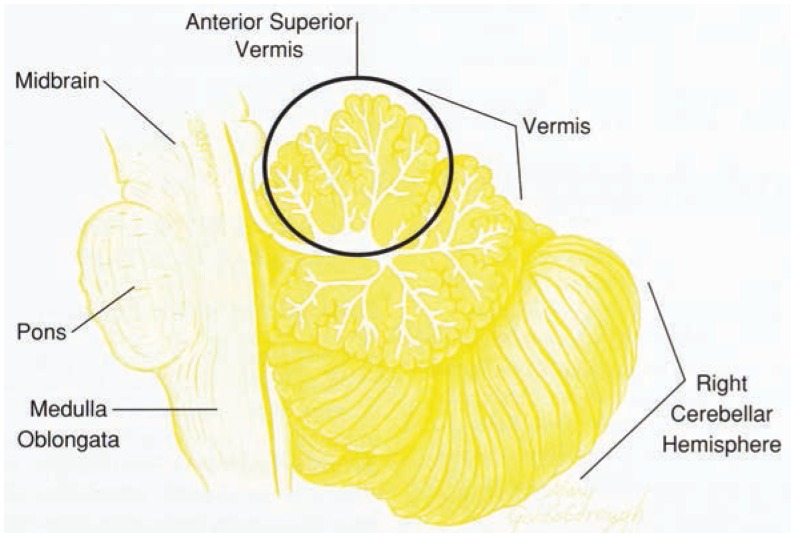
Section through the center of the cerebellum end brain stem.

The term cerebellum comes from the Latin and means “little brain.” Although it is only about one-tenth the weight of the cerebrum, it contains about as many nerve cells (approximately 5 billion for each structure) (Ghez 1991). The cerebellum consists of several subdivisions, each controlling or facilitating different behavioral functions. The cerebellum includes the left and right hemispheres and the vermis (figure 1). Unlike the cerebrum, where each hemisphere controls the opposite side of the body, in the cerebellum each hemisphere controls movement on the same side of the body. Thus, damage to the left hemisphere disrupts movement of the left arm or leg, whereas damage to the right hemisphere disrupts movement of the right limbs.

The vermis lies between and in front of the cerebellar hemispheres. It has 10 lobules and is named for its wormlike appearance. Damage to the vermis can result in poor control of posture and upright position, ataxia (widespread stance and unsteady balance), and dysarthria (irregular and explosive cadence of speech). The superior (upper) lobules of the vermis are especially involved with refinement of coordination and postural stability of the legs and trunk. The more inferior (lower) lobules support coordinated movement of the arms.

## Cognitive Functions of the Cerebellum

Nerve cells in the cerebellum communicate with nerve cells in the cerebrum, brain stem, and spinal cord, including regions involved in cognitive functions, such as spatial and other sensory perception, problem-solving, organization, and planning. Skilled performance of motor tasks involves timing, feedback from visual and sensory cues, coordination, and learned patterns or dynamics of movement that allow movements to be made quickly, smoothly, and relatively effortlessly (Gilman et al. 1981). These functions include both motor and cognitive processes. The cerebellum also participates in certain aspects of motor skill learning (Sanes et al. 1990), control of reflexive actions (e.g., blinking one’s eye in response to a signal associated with the delivery of a puff of air to the eye) (McGlinchy-Berroth et al. 1994), motor ideation (e.g., the mental representation of a movement) (Ryding et al. 1993), and tactile learning of complex figures (Roland et al. 1989).

The cerebellum may contribute to cognitive functions carried out primarily by the cerebrum. These functions include certain aspects of verbal learning (Bracke-Tolkmitt et al. 1989; Canavan et al. 1994) as well as word production (Petersen et al. 1989), problem-solving (Kim et al. 1994), and planning (Grafman et al. 1992).

## Alcohol’s Effects on Cerebellar Structure

Cerebellar degeneration is common in alcoholics (Torvik and Torp 1986; Victor and Laureno 1978). Researchers have looked at cerebellar damage in the brains of alcoholics during postmortem examination. The most consistently reported structural damage in the cerebellum of alcoholics is tissue volume loss in the anterior superior vermis (Victor et al. 1989). Tissue volume loss in this area is due especially to either shrinkage or atrophy of Purkinje cells (Charness 1993; Victor et al. 1989; Pentney 1993), large nerve cells that make up much of the volume of the vermis.

Structures at the base of the cerebellum also may be affected by excessive alcohol consumption (Allsop and Turner 1966; Victor et al. 1989). These regions regulate eye movements, particularly when both the head and the eyes are in motion. Damage to these regions can cause “slippage” of the visual image (i.e., apparent displacement of a visually perceived object) and result in visual illusions and postural instability, which may be precursors of falling (Radebaugh et al. 1985). In addition, such visual misperception can result in errors of eye-hand or eye-foot coordination, such as is needed for safe driving.

Cerebellar volume loss is confirmed by neuroimaging techniques that provide quantitative measurement of the different tissue types of the brain. Studies using computed tomography and magnetic resonance imaging (MRI)^2^ (Haubek and Lee 1979; Hillbom et al. 1986; Kennedy et al. 1976) have shown that cerebellar atrophy, shrinkage, or both can occur in the absence of clinical signs such as ataxia or clinically detectable cognitive impairment.

Cerebellar shrinkage is most notable in older alcoholics with at least a 10-year duration of alcoholism (Victor et al. 1989). Whether the degree of cerebellar shrinkage is related to the quantity of alcohol consumed is unknown. Cerebellar tissue volume also declines with age in nonalcoholics. In contrast to alcohol, which exerts its greatest effect on the anterior superior lobules, normal aging affects mostly the posterior lobules (Raz et al. 1992). Vermal shrinkage appears to be related to daily alcohol consumption but not necessarily to age in alcoholics (Karhunen et al. 1994).

## Alcohol’s Effects on Cerebellar Function

### Postural and Motor Function

The demonstration that increasing doses of alcohol are associated with increasing severity of an impairment would provide important evidence that the impairment is alcohol related. However, this type of alcohol dose-response relationship has been difficult to demonstrate for cerebellar impairment. In one study (Estrin 1987), degree of ataxia was shown not to be associated with total lifetime consumption of alcohol; however, degree of eye-foot incoordination was related to increasing alcohol consumption when ataxic alcoholics were pooled with nonataxic alcoholics and healthy control subjects.

Signs of ataxia can wax and wane corresponding with periods of alcoholic drinking and abstinence, providing evidence for specific adverse effects of alcohol on motor coordination. Abstinent alcoholics may show improvement in balance (Allsop and Turner 1966; Diener et al. 1984; Victor et al. 1989) and peripheral nerve functioning, including perception of touch and position sense, that is, the ability to detect the position of the body and its parts (Palliyath and Schwartz 1993). New research must be conducted to determine if alcohol-related imbalance results from cerebellar pathology or from poor functioning of the peripheral nervous system in the body’s extremities, such as the hands and feet. Peripheral neuropathy (i.e., the deadening of feeling in the hands and feet) can occur in alcoholics, resulting in decreased sensation that may lead to imbalance. It is unknown whether improvement in motor function results from improvement in the condition of the cerebellum or from the use of new performance strategies dependent on intact brain regions.

In one study (Diener et al. 1984), alcoholics with clinically detectable ataxia of stance and gait were tested twice: once after several hours of detoxification and again about a year and a half after the initial examination. At the second testing, some members of the group had remained abstinent from alcohol, whereas the rest had resumed drinking. Relative to initial testing, the abstainers showed marked improvement in postural stability, whereas the relapsers showed significant increase in measures of ataxia.

A functional neuroimaging study used positron emission tomography (PET) to study the ability of the brain regions to incorporate glucose, the brain’s primary energy source. This study revealed that alcoholic patients with ataxia showed hypometabolism (lower than expected glucose uptake by the brain) in the superior cerebellar vermis, whereas alcoholics without ataxia did not show cerebellar hypometabolism (Gilman et al. 1990). These results provide compelling evidence that alcohol-related ataxia is attributable specifically to cerebellar dysfunction.

Genetics also may play a role in alcohol-related motor deficits. Using PET, Volkow and colleagues (1995) examined the link between genetic predisposition to alcoholism and reduced sensitivity to alcohol in the brain. The researchers studied the effect of a drug that mimics alcohol (lorazepam) and which is believed to activate receptors in the brain that are sensitive to alcohol. Nonalcoholic subjects with family histories of alcoholism showed blunted metabolism of lorazepam in the cerebellum and smaller adverse effects on motor performance relative to nonalcoholic subjects without family histories of alcoholism. Based on these findings, it appears that a genetic predisposition to alcoholism may dampen neuronal receptor sensitivity to alcohol and reduce alcohol’s behavioral effects. These relationships also may account for the reduced untoward effect that alcohol consumption has on motor control in alcoholics with positive family histories of alcoholism, compared with those who do not have family histories of alcoholism.

Neuroimaging might help clarify the relationship between cerebellar structural and functional changes during abstinence. A similar approach has been applied to the cerebrum, where researchers have used neuroimaging techniques to track the progression and recovery of volume loss during periods of active drinking and abstinence (Shear et al. 1994*a*; Pfefferbaum in press). However, quantitative neuroimaging studies have not yet been reported on the cerebellum in recovering alcoholics.

### Cognitive Function

Alcoholics often exhibit cognitive deficits in visuospatial processing and problem-solving (reviewed in Parsons et al. 1987). These cognitive abilities, controlled primarily by the frontal and parietal lobes of the cerebrum, also may be compromised by damage to the cerebellar hemispheres. To date, however, no studies have definitely attributed cognitive dysfunction to cerebellar volume loss in alcoholics.

An MRI study identified significant cerebellar shrinkage in detoxified patients with chronic alcoholism (Davila et al. 1994). These researchers found that older alcoholics, ages 40 to 63 years, who were free of clinically detectable Korsakoff’s syndrome, had a deficit in balance and significant cerebellar shrinkage involving the hemispheres and vermis. These alcoholics were administered an extensive battery of tests that assessed cognitive and sensory as well as motor functioning. The test results revealed mild deficits in problem-solving, ability to sequence and organize information, and visuospatial capacity but did not reveal any detectable deficit in explicit memory (i.e., ability to remember new information after intervals of interference from irrelevant material) (Shear et al. 1994*b*; Sullivan et al. 1995). However, the subjects’ balance, as a group, was notably impaired, even though these alcoholics were tested after being abstinent from alcohol for about 1 month (Davila et al. 1994). The degree of vermal shrinkage in these patients was related to the extent of ataxia when balance was guided visually but did not relate to scores on the cognitive tests. Thus, cerebellar shrinkage alone could not account for these alcohol-related cognitive defects.

## Conclusion

The combined disorders of balance and visuospatial ability put alcoholics at increased risk of falls and other accidents. This may be especially true of older alcoholics (Cherpitel 1992; Malmivaara et al. 1993). Such accidents may arise from incoordination between the visual and motor systems and from poor adaptation to changes in sensory and motor input resulting from alcohol-related cerebellar damage. However, a correlation between cerebellar structural damage and the cognitive functions of the cerebellum has not been clearly demonstrated.

The reversibility of cerebellar structural changes is not known, and it is not known if the relationships observed between cerebellar damage and functional impairments persist with abstinence. To the extent that the structural change represents cell death, it may be permanent, and function may not recover. Tissue shrinkage without permanent cell loss may account for variance in the correlation between cerebellar structural and functional impairment and might represent the potential for recovery. Future investigations will need to focus on the reversibility and interrelationship of alcohol-related abnormalities in cerebellar structure and function. Results of such studies may lead to knowledge regarding the potential for recovery and guide future rehabilitation efforts.

## Footnotes

1 The exact definition of the term “alcoholism” varies with the article cited. Readers are encouraged to consult the original source for the precise definition. In general, however, alcoholism may be defined as abnormal craving for alcohol accompanied by certain behavioral manifestations including loss of control over alcohol consumption.

2 Computerized tomography and magnetic resonance imaging provide slicelike structural images of the brain, the former using X rays and the latter using radio waves within a powerful magnetic field.
